# Real-world outcomes, including intraocular inflammation, after intravitreal brolucizumab for diabetic macular edema

**DOI:** 10.1007/s10384-025-01226-y

**Published:** 2025-06-12

**Authors:** Takao Hirano, Yoshiaki Chiku, Ken Hoshiyama, Yoshiaki Takahashi, Shun Ito, Toshinori Murata

**Affiliations:** 1 Department of Ophthalmology, Shinshu University School of Medicine, 3-1-1 Asahi, Matsumoto, Nagano 390-8621 Japan; 2 Department of Ophthalmology, Omachi Municipal General Hospital, Nagano, Japan; 3 Department of Ophthalmology, Red Cross Suwa Hospital, Nagano, Japan

**Keywords:** Anti-VEGF therapy, Brolucizumab, Diabetic macular edema, Intraocular inflammation, Real-world

## Abstract

**Purpose:**

This study aimed to assess the real-world outcomes, including intraocular inflammation (IOI), following administration of intravitreal brolucizumab (Beovu 6.0 mg/0.05 mL; Novartis) injections (IVBr) for diabetic macular edema (DME).

**Study design:**

Retrospective study.

**Methods:**

A total of 56 eyes of 47 patients with DME were treated with IVBr for a minimum follow-up of at least 6 months between May 2022 and November 2023. A “non-strict pro re nata” dosing protocol for IVBr was used. The best corrected visual acuity (BCVA), central macular thickness (CMT), at baseline, 6 months, 1 year, and the latest visit date and IVBr frequency were assessed to evaluate the treatment efficacy. IOI incidence, baseline characteristics of patients with IOI, and treatment course were investigated.

**Results:**

BCVA significantly improved at 6 months and at the latest visit compared to baseline (*P* = 0.008 and 0.006, respectively). CMT was significantly thinner at 6 months, 1 year, and the latest visit compared to baseline (all, *P* < 0.001). In 46 eyes followed for more than 1 year, the number of IVBr from baseline to 1 year was 3.8 ± 1.9. Four eyes (7.1%) of four patients (8.5%) developed IOI during the observation period. All patients were women, with an average age of 70.8 ± 9.0 years (59–81 years). Upon IOI diagnosis, all patients received posterior sub-Tenon’s triamcinolone acetonide and topical betamethasone sodium phosphate, resulting in rapid resolution.

**Conclusion:**

IOI developed in four of 56 (7.1%) eyes and responded well to prompt steroid therapy after 1.5 years of IVBr use for DME. BCVA and CMT improved at all evaluation time points. With an average of 3.8 IVBr injections per year, IVBr showed long-term efficacy for DME in the real-world setting, although the occurrence of IOI should be monitored.

## Introduction

Diabetic macular edema (DME) is a leading cause of vision loss in the working population [1]. Landmark randomized clinical trials (RCTs) of ranibizumab and aflibercept report that anti-vascular endothelial growth factor (VEGF) therapy was more effective than previous mainstream focal/grid laser or topical steroid therapies in DME management [2, 3]. Anti-VEGF therapy has since become the first-line treatment for DME [4, 5]; however, extended RCTs reveal that some patients’ systems resist anti-VEGF therapy and require frequent drug administration [6, 7].

In June 2022, the anti-VEGF agent, brolucizumab, a single-chain antibody fragment, was approved in Japan for DME treatment. Two phase-3 RCTs for DME, KESTREL and KITE, show the non-inferiority of brolucizumab to aflibercept in terms of visual outcomes at 100 weeks, with fewer patients having persistent subretinal fluid (SRF) and/or intraretinal fluid (IRF) compared to aflibercept [8]. Furthermore, the early favorable therapeutic effects of brolucizumab on DME in real-world settings are reported, particularly its rapid and potent IRF/SRF suppression [9]. These results indicate that brolucizumab is expected to have a favorable therapeutic effect; however, there are not many reports on the long-term efficacy of brolucizumab in DME in clinical practice.

One concern when using a new agent in clinical practice, besides its therapeutic effect, are its possible complications. The phase-3 RCTs for neovascular age-related macular degeneration (nAMD), HAWK and HARRIER, report that the incidence of definite/probable intraocular inflammation (IOI), including retinal vasculitis and vessel occlusion, was 4.6% [10]. Furthermore, the incidence and risk factors for IOIs after intravitreal brolucizumab injections (IVBr) for AMD in the real world over a relatively long observation period are also reported [11–14]. In these studies, the incidence of IOI broadly ranged from 2.4 to 16%. In addition to the strict criteria for best corrected visual acuity (BCVA) and central macular thickness (CMT) in RCTs, patients with poor general health, such as uncontrolled blood pressure or blood glucose levels, are not eligible for enrollment. However, real-world data include patients with various conditions in the eyes or whole body, and thus have the advantage of mirroring real-world clinical practices compared to RCT data. In phase-3 RCTs for DME, IOI was reported in 3.7% of the brolucizumab 6 mg group in KESTREL and 1.7% in KITE [8]. There are objective clinical case reports on IOI after IVBr for DME [15]; however, no studies have included a large number of cases [16].

In this study, we assessed the real-world outcomes, including IOI, after IVBr for DME over a longer period.

## Subjects and methods

This retrospective observational study was approved by the Ethics Committee of Shinshu University (ID number 5820), Omachi Municipal General Hospital, and Red Cross Suwa Hospital. Due to its retrospective nature, the requirement for written informed consent was waived. Patients diagnosed with DME at Omachi Municipal General Hospital, Red Cross Suwa Hospital, and Shinshu University Hospital, who received IVBr (Beovu 6.0 mg/0.05 mL; Novartis) with a minimum follow-up of 6 months, were included in this study.

All patients underwent comprehensive ophthalmologic examination, including measurement of BCVA, slit-lamp biomicroscopy, spectral-domain optical coherence tomography (SD-OCT), and color fundus photography at each visit. SD-OCT images were captured using a 5000 high definition-OCT plus (Carl Zeiss Meditec) with a scan speed of 27,000–68,000 A-scan/s, light source wavelength of 840 nm, lateral resolution of 15 µm, and longitudinal resolution of 5 µm. SD-OCT volume images were obtained using a raster scan protocol of 512 A-scans × 128 scans/s, covering a 6 × 6 mm area centered on the fovea. Color fundus images were captured using CLARUS 500™ (Carl Zeiss Meditec AG)—a wide-field retinal imaging system covering up to 133° of the retina in a single image. Data on age, sex, ophthalmologic treatment history, estimated glomerular filtration rate (eGFR), and previous hemoglobin A1c (HbA1c; National Glycohemoglobin Standardization Program) levels were collected from medical records. The administration protocol was “non-strict pro re nata (PRN),” in which IVBr was recommended when CMT was approximately ≥ 315 μm and IRF or SRF was found on OCT at the time of the visit; it was administered whenever the patient opted for it. BCVA and CMT at baseline, 6 months, 1 year, and the latest visit date and IVBr frequency were assessed to evaluate the treatment effects. IOI incidence, characteristics of patients with IOI, and treatment course were investigated. Patients were classified into IOI and non-IOI groups, and their background clinical characteristics were compared.

## Statistics

Categorical variables are presented as numbers and percentages and were analyzed using the chi-square test. Continuous variables are expressed as mean ± standard deviation and were analyzed using paired t-test or the Mann–Whitney U test. Data analysis was performed using GraphPad Prism version 10.2.3 software (GraphPad Software). A *P* value < 0.05 was considered statistically significant.

## Results

### Baseline characteristics of the patients and their eyes

Table 1 summarizes the baseline characteristics of 56 eyes of 47 Japanese patients (19 women, 28 men) with a mean age of 68.6 ± 10.6 years. The mean HbA1c level was 7.0 ± 0.9%, and the mean eGFR was 62.0 ± 29.3 mL/min/1.73 m^2^. Of the 56 eyes, one had cataract with nuclear sclerosis (NS) 1, 10 had cataract with NS2, three had cataract with NS3, and 42 had already undergone cataract surgery with intraocular lens (IOL). Ten eyes had moderate non-proliferative diabetic retinopathy (NPDR), 31 had severe NPDR, 15 had proliferative diabetic retinopathy (PDR), and 40 had been treated with pan-retinal photocoagulation (PRP). Thirty-nine eyes were previously treated with anti-VEGF therapy for DME, and 13 eyes were previously treated with vitrectomy for diabetic retinopathy. The mean time from vitrectomy to initial IVBr was 27.8 ± 49.9 months, and the reason for undergoing vitrectomy was vitreous hemorrhage in 5 cases, epiretinal membrane in 4 eyes, and vitreomacular traction syndrome in 4 eyes. Internal limiting membrane peeling was performed in all cases that underwent vitrectomy. Anti-VEGF agents used before switching to IVBr included ranibizumab (8 eyes), aflibercept (30 eyes), and faricimab (three eyes), with a mean treatment number of 11.0 ± 13.2 before switching to IVBr.

**Table 1:** Patient demographics and baseline characteristics

Per patient	n = 47
Age, mean±SD, years	68.6 ± 10.6 (37–87)
Sex, n (female/male)	19/28
HbA1c, mean±SD, %	7.0 ± 0.9(5.0–10.2)
eGFR, mean±SD, mL/min/1.73m^2^	62.0 ± 29.3 (3–154)
Per eye	n = 56
Lens status (NS0/NS1/NS2/NS3/NS4/NS5/IOL)	0/1/10/3/0/0/42
DR severity (mild/moderate/severe NPDR/PDR), n	0/10/31/15
Previous PRP (with/without), n	40/16
Treat naïve/switching, n	17/39
History of vitrectomy (yes/no), n	13/43
Anti-VEGF agent before switching to IVBr (ranibizumab/aflibercept/faricimab), n	8/30/3
Anti-VEGF treatments before switching to IVFa, mean±SD, times	11.0 ± 13.2 (1–50)

DR, diabetic retinopathy; eGFR, estimated glomerular filtration rate; HbA1c, hemoglobin A1c; IOL, intraocular lens; IVBr, intravitreal injection of brolucizumab; N/A, not applicable; NPDR, non-proliferative diabetic retinopathy; PDR, proliferative diabetic retinopathy; PRP, pan-retinal photocoagulation; SD, standard deviation; VEGF, vascular endothelial growth factor

### Visual and anatomical outcomes

**Fig. 1:**
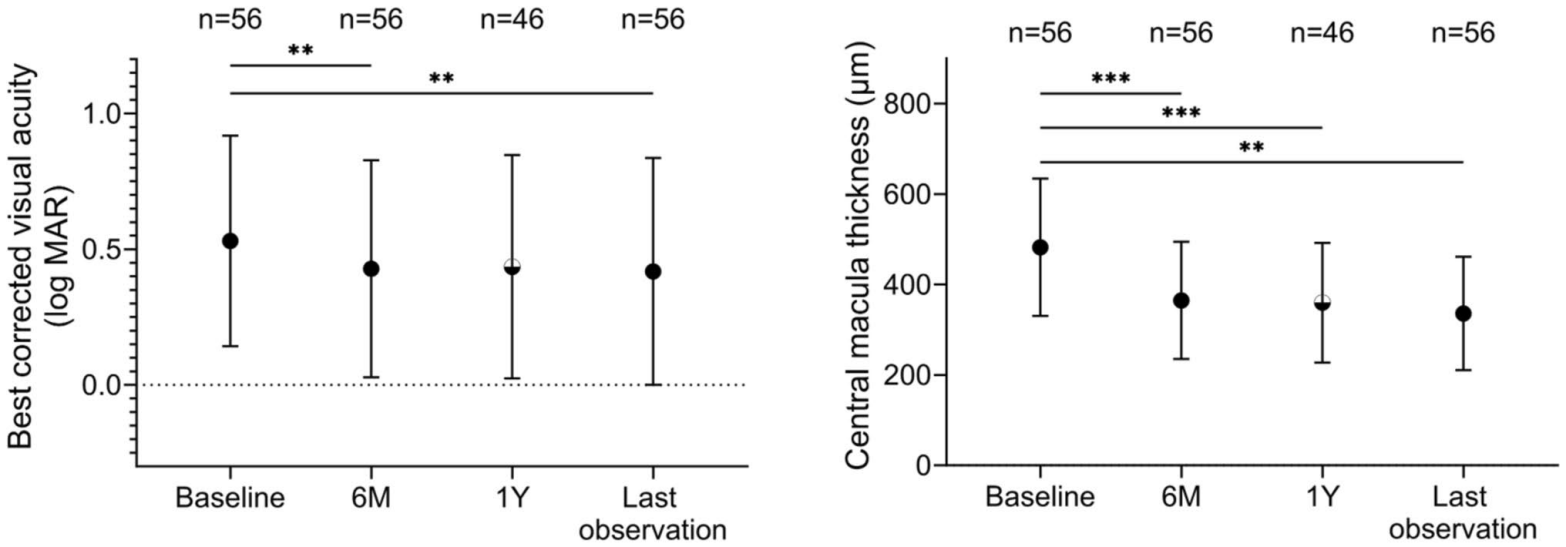
Mean change of BCVA and CMT. BCVA showed significant improvement compared to baseline at 6 months and the last observation. Compared to baseline, CMT showed significant improvement at all time points (6 months, 1 year, and the last observation). For both BCVA and CMT, the number of patients considered at baseline, 6 months, and the last observation was 56, and at 1 year, it was 46. Circles represent mean values, and bars represent standard deviations. BCVA: best corrected visual acuity, CMT: central macular thickness

Figure 1 summarizes the visual and anatomical outcomes. In all eyes (n = 56), the number of IVBr from baseline to 6 months was 2.2 ± 1.0 (in one eye the agent was changed to aflibercept due to IOI, and in one intravitreal aflibercept injection [IVA] was given. This eye was also included in the analysis.). BCVA significantly improved at 6 months (0.43 ± 0.40) compared to baseline (0.53 ± 0.39) (*P* = 0.008), and CMT was significantly thinner at 6 months (364.9 ± 129.6) compared to baseline (482.3 ± 151.8 µm) (*P* < 0.001). In the 46 eyes followed for > 1 year, the number of IVBr from baseline to 1 year was 3.8 ± 1.9 (One eye was switched to aflibercept due to IOI, and 4 IVA were administered. One eye was switched to faricimab due to IOI, and four intravitreal faricimab injections [IVFa] were administered. These eyes were included in the analysis). In cases with IOI caused by IVBr during the observation period, we switched to other anti-VEGF agents. Of note, no cases had treatment switched for reasons other than IOI. BCVA did not show statistically significant improvement at 1 year (0.43 ± 0.41) compared to baseline (0.52 ± 0.41) (*P* = 0.06); however, CMT was significantly thinner at 1 year (359.9 ± 132.4 µm) than at baseline (497.0 ± 157.0 µm) (*P* < 0.001). In all eyes (n = 56), the average number of IVBr from baseline to the latest visit (14.4 ± 5.8 months) was 4.4 ± 2.9 (One eye switched to aflibercept due to IOI, and five IVA were given. One eye switched to faricimab due to IOI, and five IVFa were given. These eyes were also included in the analysis.). BCVA showed significant improvement at the latest visit (0.42 ± 0.42) compared to baseline (0.53 ± 0.39) (*P* = 0.006), and CMT was significantly thinner at the latest visit (336.0 ± 125.3 µm) compared to baseline (482.3 ± 151.8 µm) (*P* < 0.001).

### Occurrence and characteristics of IOI

**Fig. 2:**
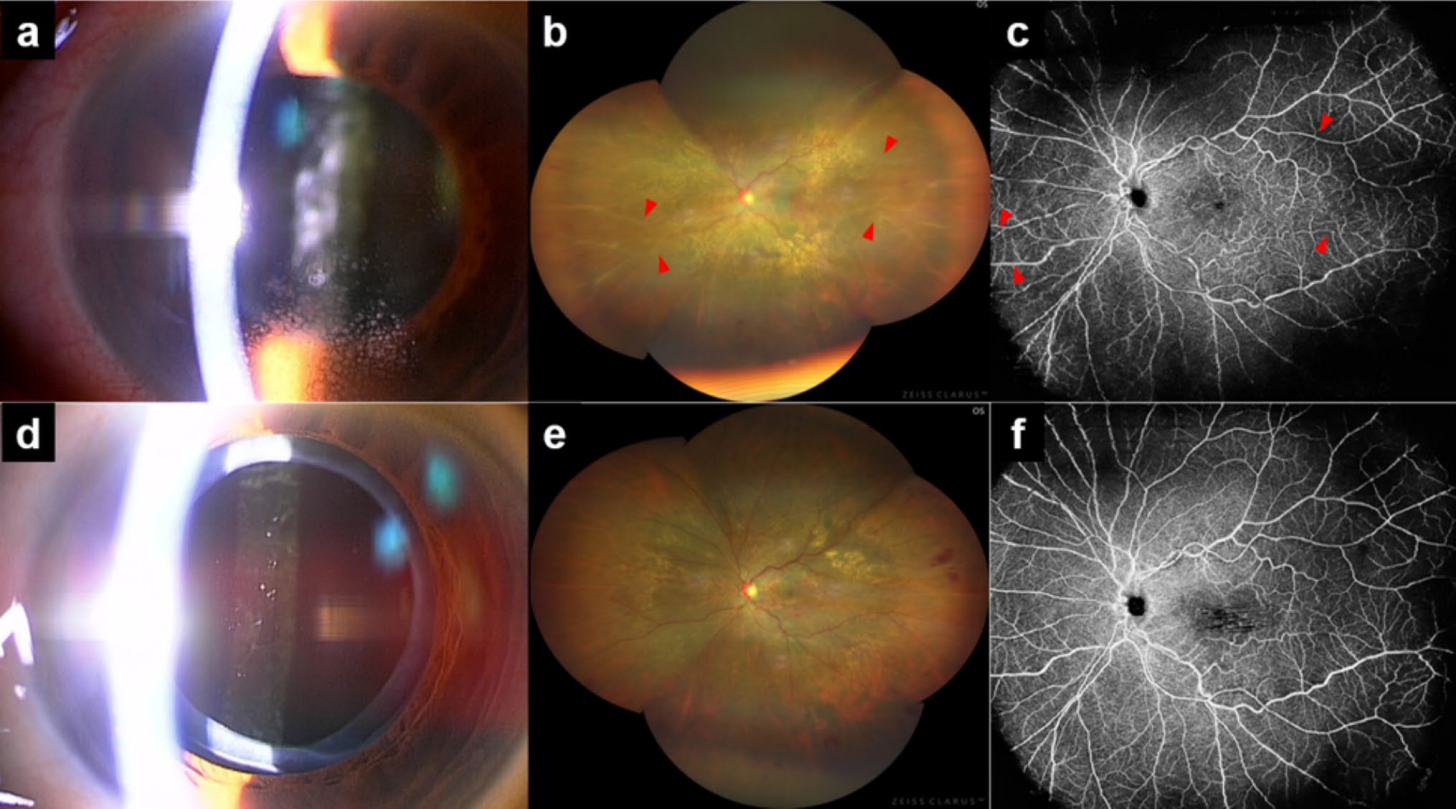
Representative case of IOI (Case No. 4). The CMT never fell below 350 μm; therefore, the patient was switched to IVBr. The patient had an unscheduled outpatient visit due to decreased visual acuity and foggy vision after the 1st IVBr. Mutton fat KP and inflammatory cells were observed in the anterior chamber and anterior vitreous (**a**). Fundus photography showed vascular white-sheathing of the peripheral blood vessels (red arrows) (**b**). Fluorescein angiography was not performed due to a history of contrast agent allergy; however, vascular occlusion was not confirmed by OCTA in the white-sheathed blood vessels (red arrows) (**c**). Inflammatory findings improved quickly after treatment with 0.1% betamethasone eye drops and STTA (20 mg/0.5 mL) injection. 6 months after the onset of IOI, although there was no inflammation (**d**–**f**), BCVA remained lower than pre-IVBr treatment due to poorly controlled IRF. BCVA: best corrected visual acuity, CMT: central macular thickness, IVBr: intravitreal brolucizumab, OCTA: optical coherence tomography angiography, IOI: intraocular inflammation, IRF: intraretinal fluid

Four eyes (7.1%) of four patients (8.5%) developed IOI during the observation period. Table 2 summarizes the clinical profiles of patients with IOI after IVBr. One eye (1.8%) had retinal vasculitis without retinal arterial occlusion. All eyes had keratic precipitates and anterior chamber cells; two had vitreous cells. All patients were women, with an average age of 70.8±9.0 years (59–81 years). All eyes had prior anti-VEGF treatment, averaging 12.8 injections (range: 2–17 injections). IOI was diagnosed at an average of 43.0 days (range: 14–91 days), with three eyes developing IOI after the first IVBr treatment and one after the second. There was no significant difference between BCVA at IOI diagnosis (0.46 ± 0.26) and baseline (0.56 ± 0.37) (*P* = 0.63). Also, no significant difference was observed between CMT at IOI diagnosis (432.5 ± 150.3 μm) and baseline (528.0 ± 143.8 μm) *(P* = 0.38). All patients received posterior sub-Tenon’s triamcinolone acetonide injections and topical betamethasone sodium phosphate. A representative case is shown in Fig. 2. After IOI was resolved, one of the four patients received five IVFa, one received five IVA until the latest visit, and two patients were followed up without any anti-VEGF therapy. No significant difference was observed between BCVA at the last observation (0.37±0.25) and baseline (0.56±0.37) (*P* = 0.50); however, in one patient, the BCVA at the last observation was worse than at the baseline due to poor CMT control. There was no significant difference between CMT at the last observation (442.3 ± 244.6 μm) and baseline (528.0 ± 143.8 μm) (*P* = 0.25). No adverse events other than IOI were identified during the observation period.

**Table 2.** Clinical profiles of patients with intraocular inflammation after brolucizumab injection

Cases	Age	Sex	Lens status	Naïve/Switch	No. of Anti-VEGF Injections before IVBr	No. of IVBr prior to IOI	IOI diagnosis fter the last IVBr days)	Anterior Chamber Inflammation	Vitreous Cells	Retinal asculitis	Retinal Vascular Occlusion	Treatment	BCVA (Snellen), CMT(μm) t the 1st IVBr	BCVA (Snellen), CMT(μm) at the diagnosis of IOI	BCVA (Snellen), CMT(μm) at the last Visit	General Conditions	HbA1c (%)
No.1	71	F	IOL	Switch	IVA: 2	2	60	1+ cells, fine KP	None	None	None	eye drops (betamethasone) STTA	20/200, 493	20/67, 565	20/40, 424	HT	6.9
No.2	59	F	IOL	Switch	IVA: 15	1	24	2+ cells, mutton fat KP	None	None	None	eye drops (betamethasone) STTA	20/29, 420	20/25, 389	20/25, 328	HT, stroke	5.9
No.3	72	F	IOL	Switch	IVA: 17	1	91	1+ cells, fine KP	2+ cells in AV	None	None	eye drops (betamethasone) STTA	20/100, 460	20/67, 239	20/50, 228	HT, HL, uterine myoma	9.8
No.4	81	F	IOL	Switch	IVR: 7, IVA: 10	1	14	1+ cells, mutton fat KP	2+ cells in AV	Yes	None	eye drops (betamethasone) STTA	20/50, 739	20/100, 537	20/100, 789	lung cancer, osteoporosis, allergy to contrast media	6.3

AV, anterior vitreous; CMT, central macular thickness, HL, hyper lipidemia; HT, hyper tension; IOI, intraocular inflammation; IVA, intravitreal injection of aflibercept; IVBr, intravitreal injection of brolucizumab; IVR, intravitreal injection of ranibizumab; KP, keratic precipitants; STTA, sub-tenon triamcinolone acetonide injection; VEGF, vascular endothelial growth factor

### Comparison of clinical characteristics between the IOI and non-IOI groups

The mean ages of patients in the IOI (four eyes of four patients) and non-IOI groups (52 eyes of 43 patients) were 70.8 ± 9.0 and 68.7 ± 10.7 years, respectively, with no significant difference between the two groups (*P* = 0.86). Furthermore, the percentages of women in the IOI and non-IOI groups were 100.0% and 30.8%, respectively; IOI occurred predominantly in the women (*P* = 0.01). There was no significant difference in HbA1c and eGFR between the IOI (6.8 ± 1.2 %, 63.5 ± 8.0 ml/min) and non-IOI groups (7.1 ± 0.9%, 63.0 ± 29.0 ml/min) (*P* = 0.41 and 0.99, respectively). The percentage of eyes with IOL in the IOI and non-IOI groups was 100% and 80.8%, respectively, with no significant difference between the two groups (*P* > 0.99). The percentage of eyes with PDR in the IOI and non-IOI groups was 0% and 28.9%, respectively; no significant difference was observed between the two groups (*P* = 0.56). The percentage of phakic eyes in the IOI and non-IOI groups was 0% and 8.7%, respectively; there was no significant difference between the two groups (*P* > 0.99). The percentage of eyes with PRP in the IOI and non-IOI groups was 50.0% and 73.1%, respectively; no significant difference was observed between the two groups (*P* = 0.57). The percentage of avitreous eyes after vitrectomy in the IOI and non-IOI groups was 25% and 23.1%, respectively, without a significant difference (*P* > 0.99).

## Discussion

We assessed the frequency and efficacy of IVBr treatment for DME for over 6 months in a real-world setting; including IOI incidence, and background clinical characteristics of patients in whom IOI occurred. At 1 year, BCVA showed no significant difference compared to baseline; however, it showed a trend toward improvement, with a significant improvement over baseline at 6 months and at the final observation.

The improvement of BCVA in 1 year was − 0.09 log MAR, converted to 4.5 letters in early treatment of diabetic retinopathy study visual acuity testing charts. On the other hand, at 1 year, the mean change in BCVA from baseline was 9.2 and 10.6 letters in KESTREL and KITE, respectively [17]. Although not easily comparable due to the different cohorts and IVBr treatment regimens used, our results are inferior to those of the representative RCTs in terms of improvement in BCVA at 1 year. The KESTREL and KITE studies include only patients who had never been treated with anti-VEGF agents in the treatment-naïve group, but this study included many eyes (n = 39, 70.0%) that had switched to IVBr because they were refractory to other anti-VEGF drugs. After vitrectomy in macaque monkeys, VEGF clearance in the vitreous was enhanced, and the half-life of bevacizumab was shortened [18]. Therefore, eyes with DME after vitrectomy might be more resistant to anti-VEGF therapy. This study included many eyes after vitrectomy (n = 13, 23.2%). The treatment-naïve eyes in this study (n = 3, 5.3%) were few; however, the mean improvement in visual acuity over 1 year was −0.30 log MAR, converted to 15 letters, which is relatively good among the whole group, supporting this notion. Another reason for the relatively poor visual acuity improvement could be the difference in the number of IVBr sessions. In this study, the average number of injections per year was 3.8, compared to 6.8 for KESTREL and 7.0 for KITE [8]. A real-world study among Japanese patients with DME suggests that the number of treatments in the anti-VEGF monotherapy group was 4.3 over two years, indicating the possibility of under-treatment [19]. Also, the administered regimen was non-strict PRN; thus, it is possible that a slightly stricter regime would have resulted in a little more improvement in visual acuity. However, in a similar real-world study of Japanese patients with DME, IVR and short-pulse focal/grid laser combination therapy improved BCVA by 4.0 letters after 6.6 IVRs in 1 year [20]. The treat-and-extend aflibercept regimen with the most extended treatment interval set to 16 weeks improved BCVA by 4.3 letters after 7.0 IVAs in 1 year [21]. Compared to these results, this study showed higher visual acuity improvement with a relatively smaller number of treatments, 4.5 letters with an average of 3.8 IVBr per year.

In terms of morphological improvement, CMT showed significant improvement over baseline at 6 months, 1 year, and the last observation. At 1 year, the proportion of patients with CMT <280 µm was 32.6%. These results were slightly poor compared to those of KESTREL and KITE (54.0% and 57.5%, respectively) [17]; however, they were considered satisfactory considering the poor baseline conditions. IVBr has shown good short-term outcomes for DME [9]; however, these results demonstrate that IVBr provides real-world visual and morphologic improvement in the long term.

The IOI incidence in this study (n = 4, 7.1%) was higher than in KESTREL (3.7%) and KITE (1.7%). The IOI incidence post-IVBr in AMD (14 of 93 [15.0%]) was higher compared to HAWK and HARRIER (4.6%). This may be because in real-world clinical practice, unlike in phase-3 clinical randomized trials, patients from various backgrounds are considered. A Japanese-only analysis of HAWK and HARRIER reports a higher IOI incidence. Also, the patients’ background and race may have influenced these results.

The characteristics of the relatively old age, female sex, and few treatments, i.e., after one or two IVBr, were consistent with those reported in patients with AMD who developed IOI [10, 12]. In this study, as in the previous report, the IOI group comprised only female patients, and although not significantly different from the non-IOI group, the mean age was higher, and the number of IVBr prior to IOI was ≤ two. A possible relationship between anti-drug antibodies after IVBr and IOIs is reported [22]. In general, sex differences in immune responses and a weaker immune system in older adults are well established facts. In the present study, IOI developed in relatively older women who had received few IVBr doses. These results suggest that the immune response could be involved in the occurrence of IOI after IVBr. The cause of post-IVBr IOI is not clear, but patients with these characteristics should be carefully monitored. In investigating other clinical factors, no significant differences in HbA1c and eGFR were observed between the IOI and non-IOI groups. There were no significant differences between the IOI group and the non-IOI group in terms of the presence or absence of IOL or PDR, whether or not PRP or vitrectomy was performed.

In this study, no cases of retinal vascular occlusion were observed, and all IOI cases improved promptly with steroid eye drops and topical administration. Oral steroids are effective in IOI with retinal vascular occlusion in AMD [23]. However, steroids are known to aggravate diabetes mellitus, and the use of oral steroids in patients with DME, IOI, and vascular occlusion may be an issue for further study. The efficacy of PRP is also reported in cases with extensive non-perfusion areas due to IOI [15]. On detecting IOI, OCT-angiography or fluorescein angiography should be used to evaluate retinal circulation. In this study, three patients with a complaint of vision loss or cloudy vision at IOI onset were promptly examined; however, one patient did not immediately consult a doctor, despite noticing foggy vision. Patients receiving IVBr should be carefully educated on IOI symptoms.

This study has several limitations. First, the sample size was small. The total number of patients who underwent IBVr and those who developed IOI were small. A multicenter study is needed to calculate IOI incidence after IVBr in patients with DME more accurately. Second, this study included only Japanese patients. The IOI incidence after IVBr in patients with AMD is reported to be higher in Japanese patients [12]. The IOI incidence in this study was also higher than the previously reported incidence and may be influenced by race. Differences in IOI incidence among racial groups may be a subject for future study.

In conclusion, this study revealed that four of 56 (7.1%) eyes developed IOI, which responded well to prompt steroid therapy after 1.5 years of IVBr use for DME in real-world clinical practice. All IOI cases occurred in women who had received IVBr once or twice. Both visual acuity and CMT showed significant improvement at the last observation compared to baseline. The average number of IVBr doses (3.8 per year) was lower than in many phase-3 RCTs. This study showed the long-term usefulness of IVBr for DME in the real-world; however, caution should be exercised regarding the occurrence of IOI.
